# Supplementary material for: Plasma Cholesterol- and Body Fat-Lowering Effects of Chicken Protein Hydrolysate and Oil in High-Fat Fed Male Wistar Rats
Source: Nutrients. 2022 Dec 16;14(24):5364. doi: 10.3390/nu14245364 (PMC9785847; doi:10.3390/nu14245364)
Supplement: Supplementary file 1 [file nutrients-14-05364-s001.zip › nutrients-1931720-supplementary.pdf]

nutrients

MDPI

Supplementary Materials

Plasma Cholesterol- and Body Fat-Lowering Effects of Chicken Protein Hydrolysate and Oil in High-Fat Fed Male Wistar Rats

Thomas A. Aloysius [1], Veronika Tillander [2], Matteo Pedrelli [2,3], Simon N. Dankel [1], Rolf K. Berge [1,4,*] and Bodil Bjørndal [1,5,*]

Supplementary tables

Table S1. Plasma lipid levels and hepatic enzyme activities in Wistar rats fed high-fat diets with different protein sources for 12 weeks[a].

	Casein	Chicken	Pork	CPH
**Plasma lipids**				
Cholesterol (mmol/L)	2.44 ± 0.47	2.19 ± 0.25	2.18 ± 0.41	1.69 ± 0.29*#
HDL cholesterol (mmol/L)	1.91 ± 0.32	1.62 ± 0.22	1.70 ± 0.34	1.32 ± 0.22*
LDL cholesterol (mmol/L)	0.49 ± 0.09	0.38 ± 0.10	0.27 ± 0.09*	0.23 ± 0.09*#
Free cholesterol (mmol/L)	0.60 ± 0.14	0.54 ± 0.08	0.54 ± 0.12	0.44 ± 0.10
Triglycerides (mmol/L)	1.02 ± 0.28	1.26 ± 0.42	1.39 ± 0.53	1.10 ± 0.32
Phospholipids (mmol/L)	2.01 ± 0.39	1.90 ± 0.26	1.90 ± 0.34	1.67 ± 0.27
Free fatty acids (mmol/L)	0.31 ± 0.08	0.28 ± 0.08	0.29 ± 0.12	0.34 ± 0.11
**Liver enzyme activity**				
β-oxidation (nmol/mg/min)	0.42 ± 0.07	0.49 ± 0.09	0.43 ± 0.09	0.49 ± 0.08
Inhibition of β-oxidation with malonyl-CoA (%)	21.9 ± 7.30	25.5 ± 4.74	20.9 ± 5.64	22.8 ± 9.30
CPT2 (nmol/mg/min)	1.07 ± 0.17	1.26 ± 0.33	1.29 ± 0.38	1.39 ± 0.29
HMG CoA synthase (nmol/mg/min)	2.94 ± 0.88	3.19 ± 0.73	3.50 ± 0.91	3.09 ± 0.74
ACOX (nmol/mg/min)	14.0 ± 2.99	14.4 ± 2.50	13.6 ± 2.27	12.2 ± 1.38
GPAT (nmol/mg/min)	1.01 ± 0.13	1.08 ± 0.13	1.09 ± 0.17	0.89 ± 0.12
FAS (nmol/mg/min)	0.21 ± 0.09	0.25 ± 0.09	0.22 ± 0.07	0.10 ± 0.04#
ACC (nmol/mg/min)	4.79 ± 2.26	4.30 ± 0.88	5.16 ± 1.43	2.72 ± 0.83
HMG CoA reductase (pmol/mg/min)	12.7 ± 2.75	13.8 ± 2.81	-	11.5 ± 2.04

Abbreviations: ACC, acetyl-CoA carboxylase; ACOX, acyl-conezyme A oxidase 1; CPT2, carnitine palmitoyltransferase II; FAS, fatty acid synthase; GPAT, glycerol-3-phosphate acyltransferase.

[a] Means ± standard deviations are shown (n = 8). Different superscript letters indicate significantly different values as determined by one-way ANOVA with Tukey's post hoc test, $p < 0.05$.
